# SOX4 promotes the growth and metastasis of breast cancer

**DOI:** 10.1186/s12935-020-01568-2

**Published:** 2020-09-29

**Authors:** Jing Zhang, Chunhua Xiao, Zhenbo Feng, Yun Gong, Baohua Sun, Zhongqi Li, Yimin Lu, Xiaojie Fei, Weizhu Wu, Xiaoping Sun, Lisong Teng

**Affiliations:** 1 Department of Surgical Oncology, The First Affiliated Hospital, School of Medicine, Zhejiang University, 79 Qingchun Road, Hangzhou, Zhejiang 310003 People’s Republic of China; 2 Division of Pathology and Laboratory Medicine, The University of Texas MD Anderson Cancer Center, 1515 Holcombe Blvd, Houston, TX 77030 USA; 3 First Department of Breast Cancer, National Clinical Research Center of Cancer, Tianjin Medical University Cancer Institute and Hospital, 1 Huan-Hu Xi Road, Ti-Yuan Bei, He Xi, Tianjin, 300060 People’s Republic of China; 4 Department of Pathology, The First Affiliated Hospital of Guangxi Medical University, No.6 Shuangyong Road, Nanning, Guangxi 530021 People’s Republic of China; 5 Department of Breast and Thyroid Surgery, Ningbo Medical Center Lihuili Eastern Hospital, Ningbo, Zhejiang 315000 People’s Republic of China

**Keywords:** Breast cancer, Growth, Metastasis, SOX4, CXCR7

## Abstract

**Purpose:**

Increasing evidence has shown that the transcription factor SOX4 is closely associated with the development and progression of many malignant tumors. However, the effect of SOX4 on breast cancer is unclear. In this study, we purposed to investigate the role of SOX4 in the growth and metastasis in breast cancer and the underlying mechanism. Moreover, the effect of SOX4 on cancer cell resistance to chemotherapeutic agents was also evaluated in vitro and in vivo.

**Methods:**

We used lentivirus technique to ectopically express SOX4 in MDA-MB-231 and SUM149 cells or knockdown SOX4 in BT474 cells, and examined the effect of these changes on various cellular functions. MTT assay was used to determine the cell viability as well as resistance to chemotherapeutic agents. The regulation of SOX4 on epithelial-mesenchymal transition (EMT)-related genes was analyzed using qRT-PCR. The binding of SOX4 to the CXCR7 gene was demonstrated using chromatin immunoprecipitation assay and dual-luciferase reporter activity assay. The effect of SOX4/CXCR7 axis on metastasis was examined using Transwell migration and Matrigel invasion assays. The expression of SOX4/CXCR7 in primary tumors and metastatic foci in lymph nodes was assessed using immunohistochemistry. Cellular morphology was investigated under phase contrast microscope and transmission electron microscopy. Moreover, the effect of SOX4 on tumor growth, metastasis, and resistance to chemotherapy was also studied in vivo by using bioluminescent imaging.

**Results:**

SOX4 increased breast cancer cell viability, migration, and invasion in vitro and enhanced tumor growth and metastasis in vivo. It regulated EMT-related genes and bound to CXCR7 promoter to upregulate CXCR7 transcription. Both SOX4 and CXCR7 were highly expressed in human primary tumors and metastatic foci in lymph nodes. Treatment of breast cancer cells with the CXCR7 inhibitor CCX771 reversed the SOX4 effect on cell migration and invasion. Ectopic expression of SOX4 increased the susceptibility of cells to paclitaxel.

**Conclusions:**

SOX4 plays an important role in the growth and metastasis of breast cancer. SOX4/CXCR7 may serve as potential therapeutic targets for the treatment. Paclitaxel may be a good therapeutic option if the expression level of SOX4 is high.

## Introduction

Breast cancer is the most common cancer and the leading cause of cancer-related death in women [1, 2], accounting for 25% of their cancers and 15% of cancer-related death [2, 3]. The cause of death by breast cancer is often not due to the primary tumor, but due to the metastasis of cancer cells to other tissues and organs. Distant metastasis usually causes the failure of clinical therapy [4]. Therefore, deep understanding of the mechanism underlying metastasis would be crucial to identify novel therapeutic targets and for the treatment of breast cancer.

SOX4 is a member of the SOX (SRY-related HMG box) gene family which consists of transcription factors involved in the development and differentiation of cells and organs, as well as the initiation and development of cancers [5, 6]. The gene of SOX4 is highly conserved in vertebrates and encodes a 47 KD protein containing 474 amino acid residues. SOX4 plays important roles in the development of bone, islet and heart, and is related to osteoporosis, cancers, and other diseases [7–12]. Previous studies have reported that SOX4 is highly expressed in over 20 malignant tumors and promotes malignant phenotypes [13, 14]. It was shown to activate CXCL12 promoter in hepatocellular carcinoma cells to modulate endothelial cell migration and angiogenesis in vivo [15]. Silencing SOX4 could inhibit the growth and metastasis of melanoma and some other tumors [7, 16–18]. These findings indicate that SOX4 functions as an oncogene. Recently, SOX4 was reported to promote epithelial-mesenchymal transition (EMT) in breast cancer [13, 19, 20]. However, the directly SOX4-regulated genes involved in the EMT process are still unknown.

Chemokines are polypeptides that regulate cell migration and serve as key regulators of metastasis. The levels of multiple chemokines are upregulated in the cell matrix of primary or metastatic tumors, compared to normal tissues [21–24]. In breast cancer cells, binding of chemokines to their receptors can activate the transcription of a series of downstream effector genes, resulting in the proliferation and metastasis of tumor cells. CXCR7, also known as RDC1, is one of the CXCR subtype cytokines. The activation of CXCR7 by its ligand CXCL12 could promote cell migration, angiogenesis, tumorigenesis, invasion, metastasis and anti-apoptosis [25]. CXCR7 is considered to be an EMT-related gene and is highly expressed in various cancer cells [26–31]. It was shown to be involved in the regulation of several pathways in cancer cells such as TGF-β1/Smad2/3 [30], PI3K/Akt [32] and β-arrestin [33] signaling pathways. However, the effect of CXCR7 on distant metastasis of breast cancer remains unclear.

In this study, we demonstrated that SOX4 promotes the growth and metastasis of breast cancer both in vitro and in vivo. SOX4 binds to the promoter of CXCR7 and upregulates its transcription. Our results indicated that SOX4/CXCR7 axis plays important roles in breast cancer and may serve as potential novel therapeutic targets in the treatment of this disease.

## Materials and methods

### Cell lines and cell culture

MDA-MB-231, BT474, SUM149, and 293T cells were purchased from American Type Culture Collection (ATCC). Cells were cultured in Dulbecco's modified Eagle's medium (DMEM, 12800-017; Gibco, Grand Island, NY, USA) containing 10% fetal bovine serum (FBS, 10099-141; Life Technologies, Carlsbad, CA, USA), 100 U/mL penicillin, and 100 mg/mL streptomycin in a humidified 5% CO_2_ atmosphere at 37 °C.

### Plasmid construction and generation of stable cell lines

The lentivirus-based vector pLOX/EW-IRES-EGFP [34] was used for ectopic SOX4-expression. SOX4 cDNA was ligated to the Sma I site of the vector and the construct was confirmed by sequencing. The SOX4-expressing vector or the control vector was co-transfected into the 293T packing cells with pAX2 and pMD2G and the lentivirus was collected for subsequent infection into MDA-MB-231 and SUM149 cells. Stably SOX4-expressing cells (MDA-MB-231-SOX4 and SUM149-SOX4) and corresponding control cells (MDA-MB-231-VECT, SUM149-VECT) were sorted using flow cytometry by BD FACS Aria (Becton Dickinson, Franklin Lakes, NJ, USA). For SOX4 knockdown, lentivirus-based pLKO.1-TRC-puro constructs containing a SOX4 short hairpin RNA (shRNA NT or #13-#17) were purchased from Open Biosystems (Huntsville, AL, USA). The lentivirus was prepared as above and used to infect BT474 cells. Stably SOX4-knockdown cells (BT474-14, BT474-16) and control cells (BT474-NT) were yielded after treatment with puromycin (Sigma, San Francisco, CA, USA) for 14 days. To introduce luciferase into tumor cells for bioluminescent imaging in vivo, pMig-Luc-mCherry, pUMVC and pMD2G were co-transfected into the 293T packing cells and the retrovirus was collected to infect tumor cells for xerograph experiments.

### Western blot

The protein lysate was resolved by SDS-PAGE and transferred to PVDF membrane. After blocking with 5% bovine serum albumin (BSA), the membrane was incubated with the primary antibody overnight at 4 ºC, followed by incubation with the secondary antibody at room temperature for 1 h. The antibodies used in this study included anti-SOX4 (Cat#H00006659-A01, Abnova, Taipei), anti-GAPDH (Cat#MA5-15738, Invitrogen, Grand Island, NY, USA), horseradish peroxidase (HRP)-conjugated goat anti-mouse IgG (H&L) (Cat#G21040, Invitrogen), and HRP-conjugated goat anti-rabbit IgG (H&L) (Cat#31460, Invitrogen).

### MTT assay

Cells were seeded in 96-well plates at a density of 6 × 10^6^ cells per well and incubated at 37 ºC in humidified 5% CO_2_ for 24 h. The cells were stained with MTT (Cat#CT02, Sigma) at each time point for 1 h at 37 °C. The absorbance was measured at 490 nm using a spectrophotometer (Bio-Rad, Hercules, CA, USA). For the analysis of resistance to chemotherapeutic agents, carboplatin, adriamycin, 5-fluorouracil, or paclitaxel at different concentrations was added at the beginning of the cell culture which was carried out for 48 h.

### Transwell assay

Migration was measured using Matrigel-free Transwell plates (Corning, Midland, MI, USA) with an 8 μm porous membrane. For invasion assay, membrane of the upper chambers was coated with Matrigel (BD Biosciences, Sparks, MD, USA) before use. In total, 1 × 10^5^ cells were plated in the upper chambers of the Transwells. After a 24-h incubation, migrating or invading cells were stained with 0.5% crystal violet, then photographed at 200× and counted in five random fields. CXCR7 inhibitor CCX771 was a courtesy from ChemoCentryx (Mountain View, CA, USA).

### RT-qPCR

Total RNA was prepared with the Trizol reagent according to the manufacturer’s instructions (Invitrogen). cDNA was synthesized using SuperScript Kit (Invitrogen). Real-time PCR was performed using SYBRH Select Master Mix for CFX (Invitrogen). Relative quantification was achieved by normalization to the amount of GAPDH control RNA. The primers used are shown in Table 1.

**Table 1:** Primers for qPCR

SOX4-F	5′-GACATGCACAACGCCGAGATCT-3′
SOX4-R	5′-GTAGTCAGCCATGTGCTTGAGG-3′
CXCR7-F	5′-CCAAGACCACAGGCTATGACAC-3′
CXCR7-R	5′-TGGTTGTGCTGCACGAGACTGA-3′
Vimentin-F	5′-CACAAGCAGAGTGCTGAAGGTG-3′
Vimentin-R	5′-ATCTGGCGTTCCAGGGACTCAT-3′
N-Cadherin -F	5′-AGACGCTAGTGGAGGAGTGCAA-3′
N-Cadherin -R	5′-GTAGGATCTCCGCCACTGATTC-3′
E-Cadherin-F	5′-GCCTCCTGAAAAGAGAGTGGAAG-3′
E-Cadherin-R	5′-GGAGATACCAGTTCCACAGGTC-3′
Fibronectin-F	5′-ACAACACCGAGGTGACTGAGAC-3′
Fibronectin-R	5′-GGACACAACGATGCTTCCTGAG-3′

*F* forward, *R* reverse

### Chromatin immunoprecipitation assay

Cultured MDA-MB-231-VECT and MDA-MB-231-SOX4 cells were treated with 1% formaldehyde at 37 °C for 15 min, and lysed with nuclei-swelling buffer. After sonication and centrifugation, cytoplasmic extract was incubated at 4 °C overnight with 5 mg of anti-SOX4 (Cat#ab86809, Abcam, Cambridge, UK) or nonspecific IgG control antibody. Cytoplasmic extract not incubated with antibody was saved as an input sample. The antibody-bound complex was precipitated by protein A-Sepharose beads (Amersham Biosciences, Little Chalfont, Buckinghamshire, UK). The beads were washed and the protein-DNA complex was eluted from the beads with 250 ml of elution buffer (1% SDS and 0.1 M NaHCO_3_) at 37 °C for 15 min. The DNA in the immunoprecipitated complex and the DNA in the previously saved input fraction were released by incubation at 65 ℃ for 2 h with 200 nM NaCl and 20 mg of proteinase K. DNA was amplified by PCR under the following conditions: 96 °C for 15 s, 54 °C for 30 s and 72 °C for 30 s, and 40 cycles. The primers used are showed in Table 2.

**Table 2:** Primers used in chromatin immunoprecipitation assay

CXCR7-F	5′-GAAGAGGCATTCACAGGAGC-3′
CXCR7-R	5′-CAGAAAGGAGCCTCTAGC-3′

*F* forward, *R* reverse

### Dual-luciferase reporter activity assay

The plasmid pGL3-CXCR7 containing the CXCR7 promoter upstream of the firefly luciferase reporter gene and the corresponding empty vector were purchased from HIBIO (Hangzhou, China). MDA-MB-231-VECT and MDA-MB-231-SOX4 cells were transfected with pGL3-CXCR7 and empty vector, respectively, when the cells grew to 60%-80% confluence. Next day, the cells were lysed and the luciferase activity was tested using the Dual-Luciferase Reporter Assay System (Promega, Madison, WI, USA). The activity was calibrated to Renilla luciferase activity. Each treatment was carried out in triplicate.

### Immunohistochemistry (IHC)

The tissue chip slide (ZL-BRM961, SUPERBIOTEK, Shanghai, China), which contained tissues of primary breast carcinomas and corresponding lymph node metastases (n = 36), was deparaffinized in xylene and rehydrated in graded alcohol solution, followed by treatment with 3% H_2_O_2_ to quench the endogenous peroxidase for 15 min and 1% BSA for 1 h at room temperature to block the potential nonspecific binding. The slides were then incubated with primary monoclonal antibody against SOX4 (Cat#ab237903, Abcam) or CXCR7 (Cat#ab38089, Abcam) overnight at 4 °C prior to incubation with biotinylated secondary antibody for 20 min at room temperature. Finally, the slides were treated with avidin–biotin complex reagent and stained with 3, 3 -diaminobenzidine according to manufacturer's protocol. Staining results were analyzed for the staining intensity as well as the percentage of positive cells. The staining intensity was scored 0 point for no obvious coloring, 1 point for mild, 2 points for moderate, or 3 points for strong. The percentage of positive cells for each slide was calculated from 3 different microscopic fields (200x) and was scored as follows: 0 to 5%, 0 point; 6% to 25%, 1 point; 26% to 50%, 2 points; 51% to 75%, 3 points; and > 75%, 4 points. The overall score was the sum of the staining intensity and the percentage of positive cells: 0, negative; 2 to 3, weakly positive; 4 to 5, moderate; and 6 to 7, strongly positive.

### Tumor xenograft

Six-week-old male mice with severe combined immunodeficiency (SCID) were obtained from Jackson Laboratory (Bar Harbor, ME, USA). Cells (5 × 10^6^) expressing luciferase were injected into the left mammary fat pad of each mouse. The tumors were examined weekly using bioluminescent imaging after intraperitoneal injection of 150 mg/kg D-luciferin (Caliper LifeSciences, Hopkinton, MA, USA). The length (a) and width (b) of the tumors were also recorded every week. The tumor volume was calculated by the formula of V = ab^2^/2 mm^3^. In the study of metastasis, tumors were removed when their sizes exceeded 2 cm × 2 cm. Metastasis was followed by bioluminescent imaging. By the end of the 16th week, mice were euthanized, dissected and examined for organ metastases. In the study of resistance to paclitaxel, 3 weeks after cell injection, i.e., day 0 of treatment, the tumor-bearing mice were administered with paclitaxel at a dose of 10 mg/kg or the same volume of phosphate buffered saline (PBS). The treatment was repeated at day 7 and the tumors were examined using bioluminescent imaging at day 14.

## Results

### *SOX4 promotes breast cancer cell proliferation both *in vitro* and *in vivo

**Fig. 1:**
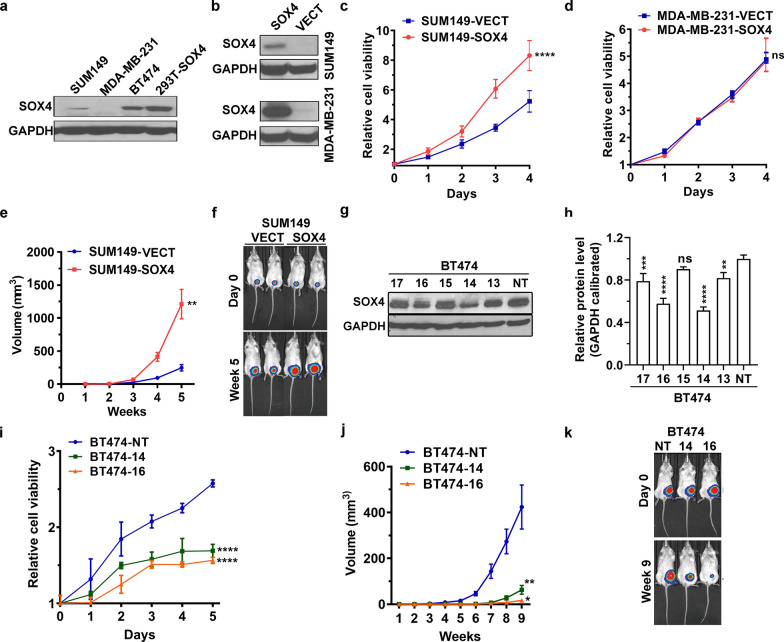
SOX4 promotes breast cancer cell proliferation both in vitro and in vivo. **a** Western blot analysis of SOX4 expression in three human breast cancer cell lines. Embryonic kidney cell line 293 T with ectopic SOX4 expression (293 T-SOX4) was used as positive control. GAPDH was used as loading control. **b** Western blot showing SOX4 protein levels in MDA-MB-231-SOX4 cells and SUM149-SOX4 cells as well as the control cells. GAPDH was used as loading control. **c** and **d** MTT assay showing cell viability in SOX4-expressing cell lines compared with control. The data are presented as the mean ± s.d. of sextuplicate wells. Unpaired two-tailed *t*-test: ***p < 0.001. **e** In vivo tumor growth from SOX4-expressing cells and control cells. 5 × 10^6^ cells were injected into the mammary fat pad of SCID mice. The length a and width b of tumors were recorded weekly. The tumor volume was calculated by the formula of V = ab^2^/2 (mm^3^). The data are presented as the mean ± s.e.m. of 6 mice. Unpaired two-tailed *t*-test: **p < 0.01. **f** Bioluminescent imaging illustrating tumor growth in vivo. **g** Western blot showing the SOX4 expression in BT474 cells with individual shRNAs. **h** Semi-quantitative analysis of western blotting. Unpaired two-tailed *t*-test: **p < 0.01, ***p < 0.001, ****p < 0.0001. **i** MTT assay showing decreased cell viability in SOX4-knockdown cells (BT474-14, BT474-16) compared to the control (BT474-NT). **j** Slower in vivo tumor growth from SOX4 knockdown cells compared to the control cells at different time points. The data are presented as the mean ± s.e.m. of 5 mice. **k** Bioluminescent imaging showing slower tumor growth with SOX4 knockdown

The protein expression levels of SOX4 in breast cancer cell lines SUM149, MDA-MB-231, and BT474 were variable, low in SUM149 and MDA-MB-231 and high in BT474 by Western blot (Fig. 1a). Thus, we chose SUM149 and MDA-MB-231 for ectopic SOX4 expression which was successfully achieved using lentivirus carrying the SOX4 gene (Fig. 1b), with the SOX4 expression levels significantly higher in MDA-MB-231-SOX4 cells than those in SUM149-SOX4 cells.

MTT assay showed that SUM149 cells with ectopic SOX4 expression had higher viability than the control, though no difference in viability was observed between the MDA-MB-231-SOX4 cells and their control cells (Fig. 1c and 1d). To confirm the in vivo findings, we injected the SUM149-SOX4 and SUM149-VECT cells into the mammary fat pad of SCID mice. As shown in Fig. 1e, the tumor volume in the SUM149-SOX4 group was bigger than that in the SUM149-VECT group at all the post-injection time points, which was consistent with the bioluminescent imaging results (Fig. 1f).

On the other hand, we knocked down SOX4 using lentivirus-mediated SOX4 shRNA in BT474 cells whose SOX4 expression was the highest among the cell lines tested. Stable SOX4 knockdown was achieved with shRNA-14 and shRNA-16 (Fig. 1g and h). MTT assay reveled that SOX4 knockdown reduced the viability of BT474 cells (Fig. 1i). Likewise, our in vivo experiments also showed slower growth in the xenografts with SOX4 knockdown (Fig. 1j and k).

### *SOX4 enhances cancer cell metastasis both *in vitro* and *in vivo

**Fig. 2:**
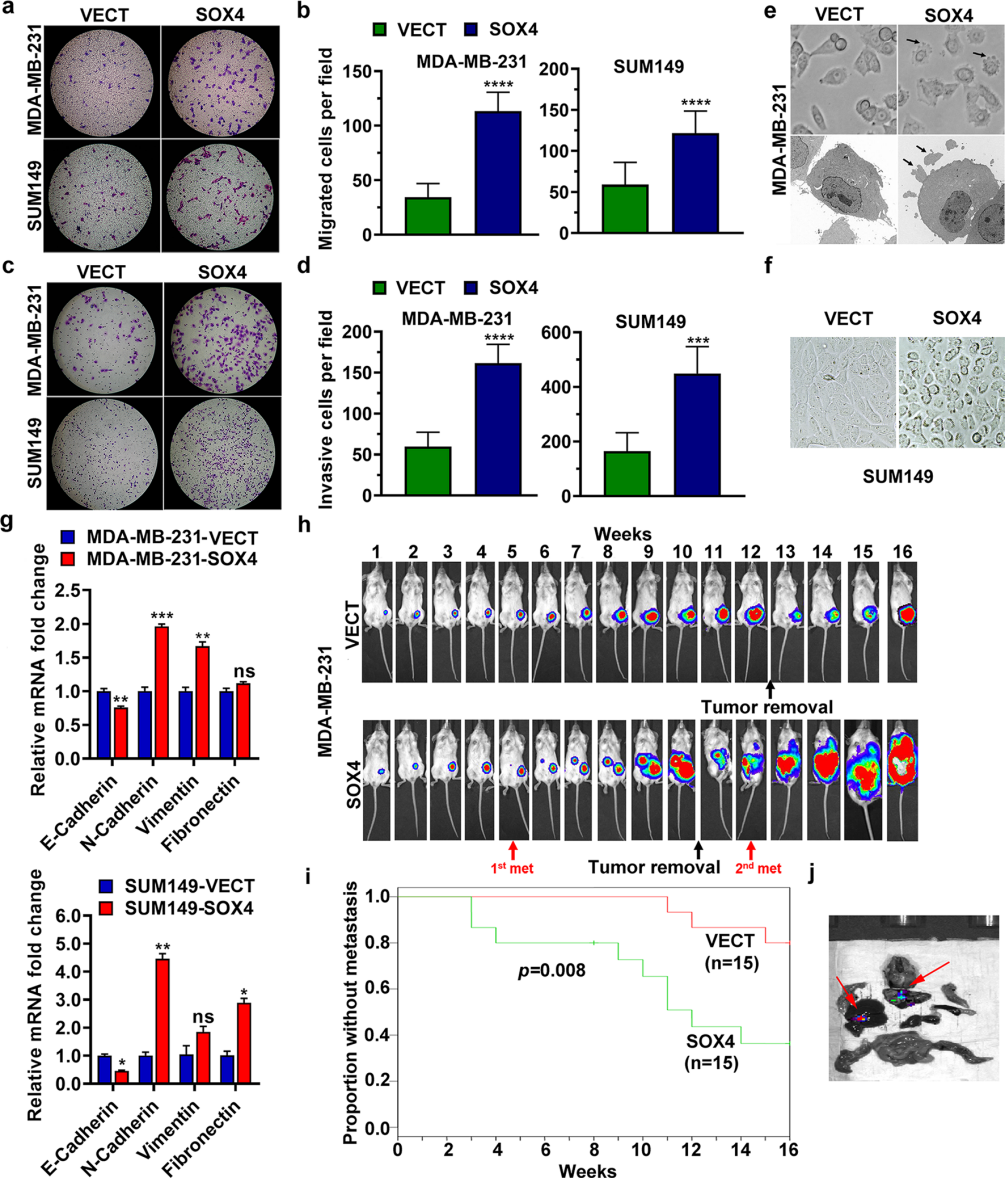
SOX4 enhances the cancer cell metastasis both in vitro and in vivo. **a** Photographs showing Transwell migration assay. **b** Histograms of the Transwell migration assay results. The data are presented as the mean ± s.d. of triplicate wells. Unpaired two-tailed *t*-test: *p < 0.05. **c** Photographs showing Matrigel invasion assay. **d** Histograms of the Matrigel invasion assay results. The data are presented as the mean ± s.d. of triplicate wells. Unpaired two-tailed *t*-test: *p < 0.05, ***p < 0.001. **e** Phase contrast (upper) and electronic microscopic (lower) photographs showing pseudopodia (arrows) in MDA-MB-231 cells. **f** Phase contrast photographs showing intercellular space in day-4 culture of SUM149 cells. **g** qRT-PCR analysis of EMT-related genes in MDA-MB-231 (upper panel) and SUM149 (lower panel) cells. The data are presented as the mean ± s.d. of triplicate wells. Unpaired two-tailed *t*-test: *p < 0.05, **p < 0.01, ***p < 0.001. **h** Bioluminescent imaging showing tumor growth and metastasis in vivo. 5 × 10^6^ cells (MDA-MB-231-VECT or MDA-MB-231-SOX4) were injected into the mammary fat pad of SCID mice. The primary tumors were removed when their size exceeded 2 cm × 2 cm. **i** Kaplan and Meier analysis of tumor metastasis for 15 pairs of mice. **j** Bioluminescent image for metastasis in various organs of a mouse with SOX4-expressing tumor

We studied the effect of SOX4 on migration and invasion of breast cancer cells using Transwell migration assay and Matrigel invasion assay, respectively. Ectopic SOX4 expression in SUM149 and MDA-MB-231 cells significantly increased cell migration and invasion (Fig. 2a to d). Interestingly, we observed morphological changes in SOX4-expressing MDA-MB-231 cells which showed more pseudopodia than did the control cells (Fig. 2e). Moreover, there were more intercellular spaces in cultured SUM149 cells with SOX4 expression than in the control cells (Fig. 2f). Since EMT is considered to be an essential change during metastasis, we then evaluated the expression of EMT-related genes *E-cadherin, N-cadherin, Vimentin, and Fibronectin,* using qRT-PCR [35–38]. Our results showed that, in both MDA-MB-231 cells and SUM149 cells, SOX4 expression reduced the mRNA level of E-cadherin, an epithelial marker, but increased the levels of the three mesenchymal markers, Vimentin, N-cadherin, and Fibronectin (Fig. 2g).

We further investigated the effect of SOX4 on metastasis in vivo by examining metastasis in tumor bearing mice. We chose MDA-MB-231 cells to study the effect of SOX4 on metastasis in vivo with the view that the observed effect on metastasis would not have been due to an advantage in cell viability since there was no significant increase in cell viability of these cells compared to that of the control cells (Fig. 1d). A representative pair of mice was shown in Fig. 2h; bioluminescent imaging first detected metastasis at the 5th week in the mouse with a tumor derived from the MDA-MB-231-SOX4 cells. After the primary tumor was removed due to oversize, a second metastasis was detected at the 12th week. In contrast, no metastasis was seen in the control mouse with an MDA-MB-231-VECT tumor during the whole period of this experiment. A total of 15 pairs of mice was tested and the time to metastasis was much shorter in the SOX4-expression group than in the control group (p = 0.008, log-rank test, Fig. 2i). Finally, mice were euthanized at the end of 16th week and metastases in various organs were examined by bioluminescent imaging (Fig. 2j). Lung metastases was seen in 87% (13/15) of mice in the group with ectopic SOX4-expression, but only in 47% (7/15) of mice in the control group (*p* = 0.05, Fisher’s exact test). Liver metastasis was found in 20% (3/15) of mice in the SOX4-expressing group, whereas no liver metastases was observed in the control group.

### SOX4 binds to the promoter of the CXCR7 gene and activates its transcription

**Fig. 3:**
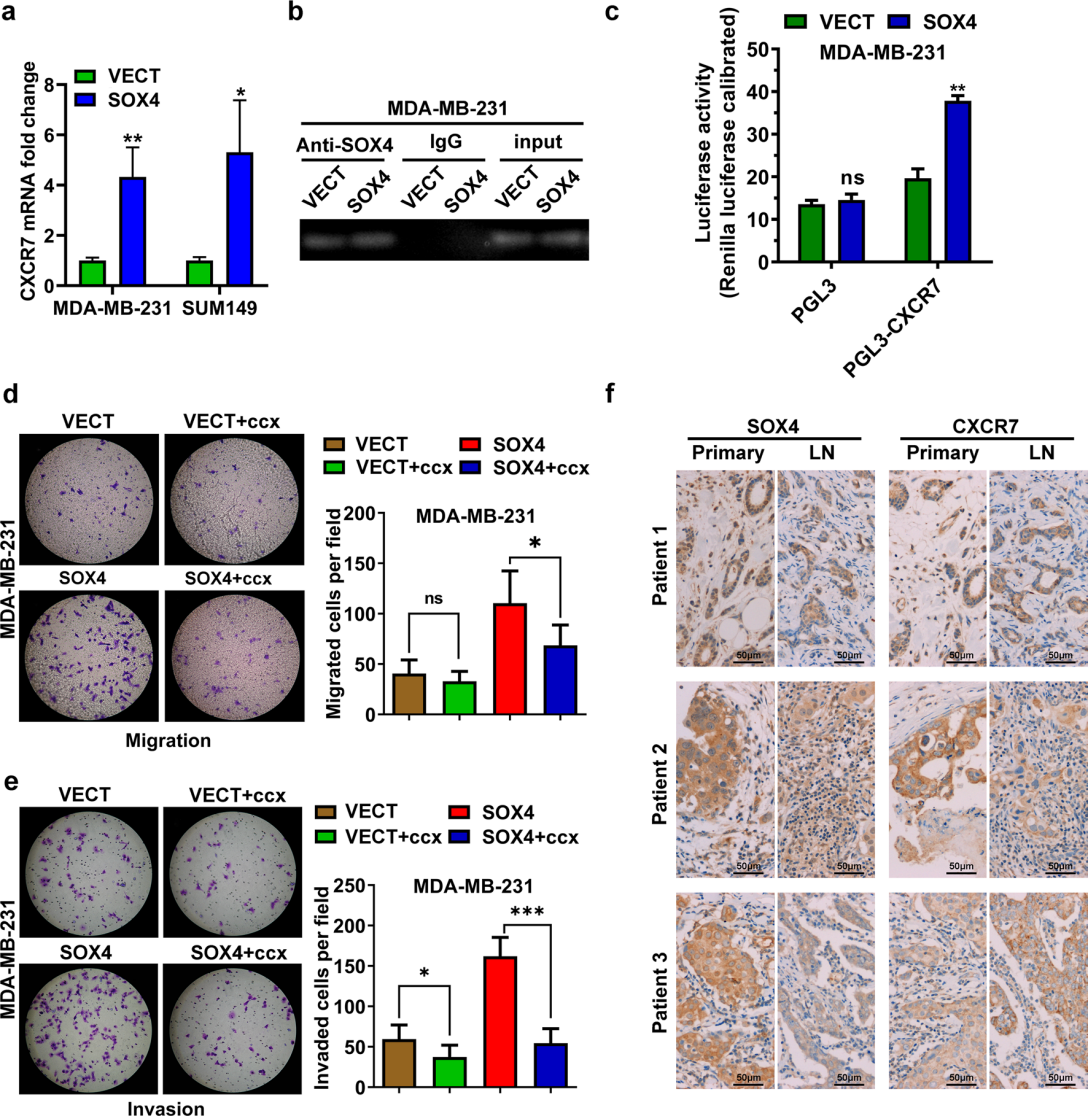
SOX4 binds to the promoter of the CXCR7 gene and activates its transcription. **a** Increased amount of CXCR7 mRNA in SOX4-expressing MDA-MB-231 and SUM149 cells. The data are presented as the mean ± s.d. of triple wells. Unpaired two-tailed *t*-test: *p < 0.05, **p < 0.01. **b** CXCR7 gene fragments amplified from pull-down samples of the chromatin immunoprecipitation assay using the SOX4 antibody and non-specific IgG, and the input samples. **c** Dual-luciferase reporter assay showing increased CXCR7 promoter-specific reporter activity associated with SOX4 overexpression. **d** and **e** CXCR7 inhibitor CCX771 (ccx) reversed the enhancing effect of SOX4 on migration and invasion. The data are presented as the mean ± s.d. of triplicate wells. Unpaired two-tailed *t*-test: *p < 0.05, ***p < 0.001. **f** IHC analysis of SOX4 and CXCR7 expression in human breast primary tumors (Primary) and lymph node metastatic foci (LN)

By using gene expression microarray, we found that the expression of CXCR7 was significantly upregulated in SOX4-expressing cells, which was confirmed by qRT-PCR (Fig. 3a). Examination of the CXCR7 gene promoter revealed two DNA sequences, AACAAAG and TACAAAG, which are consistent with the consensus SOX4 binding motif A/T A/T CAA A/T G [39]. We then used chromatin immunoprecipitation assay and amplified, with primers for CXCR7, a 384 bp fragment from MDA-MB-231 cell DNA co-precipitated with the SOX4 antibody (Fig. 3b). No amplified fragment was obtained when non-specific IgG was used as the capture antibody. Moreover, the dual-luciferase reporter assay showed that ectopic SOX4 expression significantly increased the activity of CXCR7 promoter (Fig. 3c). We further found that CXCR7 inhibitor CCX771 was able to reverse the enhanced cell migration and invasion due to ectopic SOX4 expression (Fig. 3d and e). These results indicated that SOX4 directly binds to the promoter of the CXCR7 gene and upregulates its transcription, which might result in an increased metastatic capacity.

We performed IHC and detected the SOX4 and CXCR7 expression in all the 36 pairs of human primary breast carcinomas and their corresponding lymph node metastases in a tissue chip slide (Fig. 3f). The expression of SOX4 and CXCR7 was strongly positive in 28/36 and 23/36 of the primary tumors and in 27/36 and 23/36 of the metastatic lymph nodes, respectively, although the IHC staining intensity was not significantly different between the primary tumors and lymph node metastases..

### SOX4 increases the susceptibility of breast cancer cells to paclitaxel

**Fig. 4:**
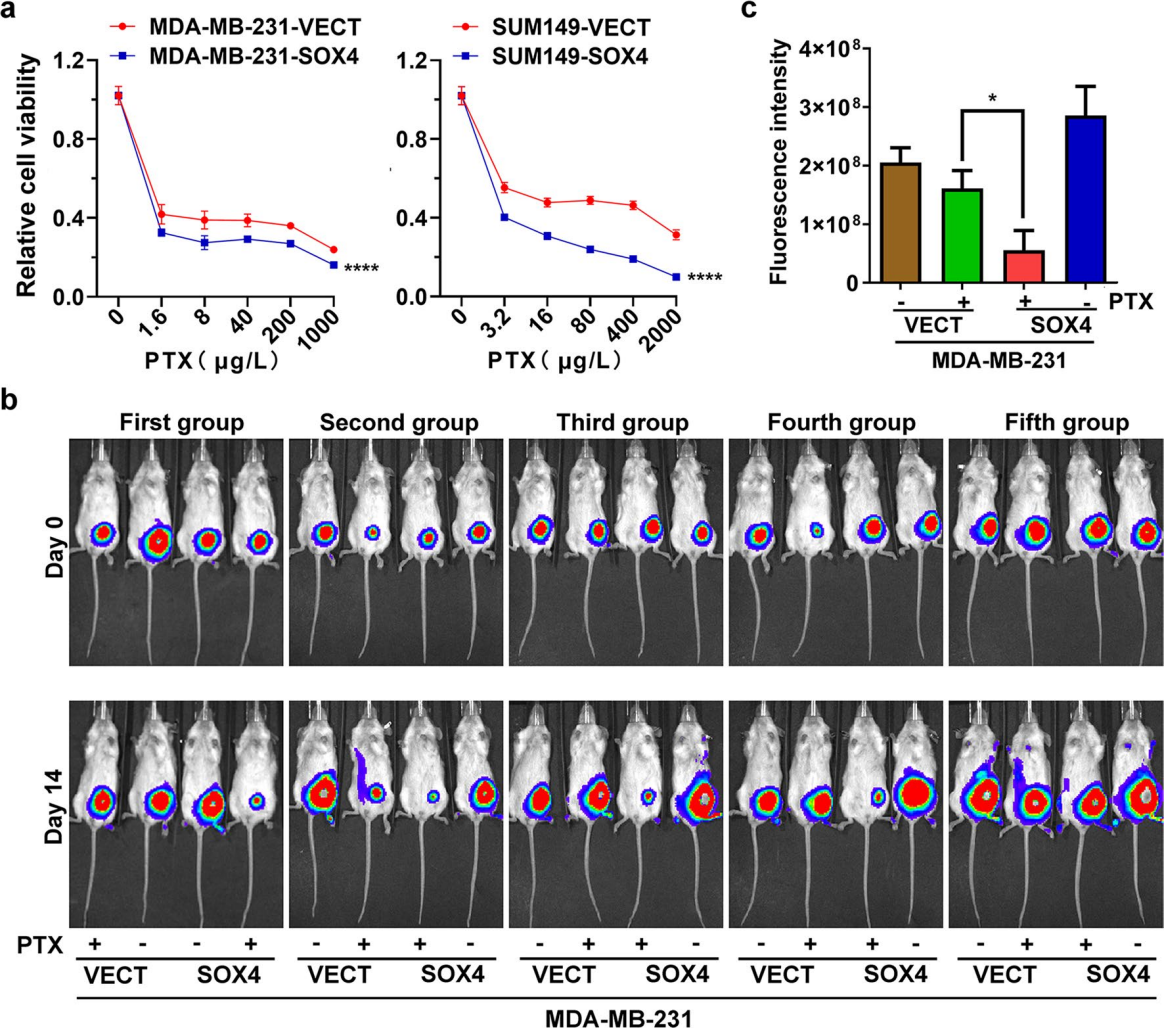
SOX4 increases the susceptibility of breast cancer cells to paclitaxel. **a** MTT assay showing cell viability in response to paclitaxel (PTX) after 48 h treatment. The data are presented as the mean ± s.d. of sextuple wells. Unpaired two-tailed *t*-test: ***p < 0.001. **b** Bioluminescent imaging of tumor growth 14 days after treatment with Paclitaxel. 5 × 10^6^ cells (MDA-MB-231-VECT or MDA-MB-231-SOX4) were injected into the mammary fat pad of SCID mice in 5 groups. Three weeks later (day 0), the tumor-bearing mice were treated with paclitaxel (PTX) or PBS. The treatment was repeated at day 7 and the tumors were examined using bioluminescent imaging at day 14. **c** Statistical analysis of results in **b**. The data are presented as the mean ± s.d. of 5 mice. Unpaired two-tailed *t*-test: *p < 0.05

To explore the potential role of SOX4 in breast cancer treatment, we investigated its effect on cancer cell resistance to several commonly used chemotherapeutic agents including ecarboplatin, adriamycin, 5-fluorouracil, and paclitaxel. There was no significant difference in response to ecarboplatin, adriamycin, or 5-fluorouracil between SOX4-expressing cells and the control cells in both MDA-MB-231 and SUM149 (data not shown). However, we observed that SOX4 overexpression significantly increased the susceptibility of MDA-MB-231 and SUM149 cells to paclitaxel (Fig. 4a). On the contrary, SOX4 knockdown increased the IC_50_ (Additional file 1: Figure S1). In addition, bioluminescent imaging at day 14 showed that treatment with paclitaxel suppressed the tumor growth in vivo (Fig. 4b). Moreover, the fluorescence intensity reduced greater in the SOX4-expression group than the control group (Fig. 4c), indicating that SOX4 increased the susceptibility of MDA-MB-231 cells to paclitaxel.

## Discussion

Breast cancer is the most prevalent cancer and the leading cause of cancer-related death in women [1, 40]. Distant metastasis usually leads to the failure of clinical therapy. In this study, we found that transcription factor SOX4 promotes tumor growth and metastasis, likely through binding to the promoter of CXCR7 gene and activating its transcription. CXCR7 was reported to enhance the growth of MDA-MB-435 breast cancer cells, and the proliferation as well as metastasis of lung cancer cells [26]. It was also found to facilitate breast cancer cells to cross the blood–brain barrier, leading to brain metastasis [41]. Our results suggested that CXCR7 is a downstream gene of SOX4. Importantly, as shown by our IHC studies, both SOX4 and CXCR7 are strongly expressed in human primary breast cancer cells and the metastatic foci in lymph nodes. Moreover, treatment of MDA-MB-231 cells with CCX771, a small molecule inhibitor of CXCR7, could effectively reverse the enhanced migration and invasion due to ectopic expression of SOX4. These findings suggested that inhibition of SOX4/CXCR7 pathway may reduce distant metastasis in the treatment for human breast cancer.

Interestingly, cells with ectopic SOX4 expression have more pseudopodia and maintain more intercellular space in culture, changes that are suggestive of EMT. To date, it is well accepted that EMT phenomenon is the favored explanation of distant metastasis of breast cancer [42]. EMT enhances cell motility and helps the release of breast cancer cells from the primary tumor and the establishment of metastatic colonies at distant sites [43]. Previous studies have reported that CXCR7 promotes EMT by upregulating TGF-β1 [29–31]. We determined the expression levels of EMT-related genes in SOX4-expressing cells and found that the expression of three mesenchyme markers Vimentin, N-cadherin, and Fibronectin were upregulated, and that of the epithelial marker E-cadherin was downregulated. These findings indicated that SOX4/CXCR7 axis may promote EMT and play an important role in the metastasis of breast cancer.

One of the important findings in this study is that ectopic expression of SOX4 increases the susceptibility of breast cancer cells to paclitaxel, suggestive of the potential use of paclitaxel in breast cancer treatment if the expression level of SOX4 in the tumor is high. Of note, the MDA-MB-231 and SUM149 cells we tested are triple negative breast cancer cells. Currently, compared to hormonal receptor positive and HER2 positive breast cancers, triple negative cancers lack effective treatment [44]. Our finding may provide a new therapeutic strategy for triple negative breast cancer based on the expression of SOX4. Nevertheless, further studies are required to investigate the status of SOX4 expression in triple negative tumors and to directly test the efficacy of Paclitaxel for their treatment.

In conclusion, our study revealed that SOX4 promotes the growth and metastasis of breast cancer. SOX4/CXCR7 may serve as potential therapeutic targets for the treatment of breast cancer. Paclitaxel may be a good therapeutic option if the expression level of SOX4 in the tumor is high.

## Supplementary information

**Additional file 1: Figure S1. **SOX4 knockdown increases IC_50_ of paclitaxel in BT474.

## Data Availability

Not applicable.
