# Supplementary figures and images for: SOX4 promotes the growth and metastasis of breast cancer
Source: Cancer Cell Int. 2020 Sep 29;20:468. doi: 10.1186/s12935-020-01568-2 (PMC7523060; doi:10.1186/s12935-020-01568-2)

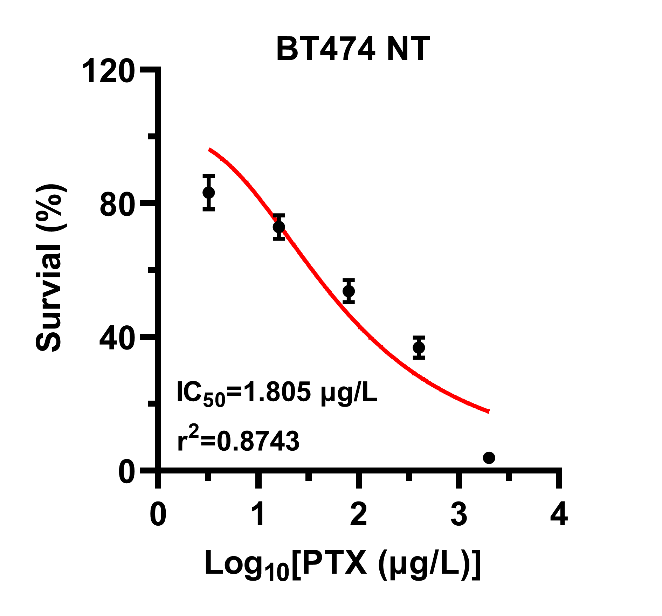

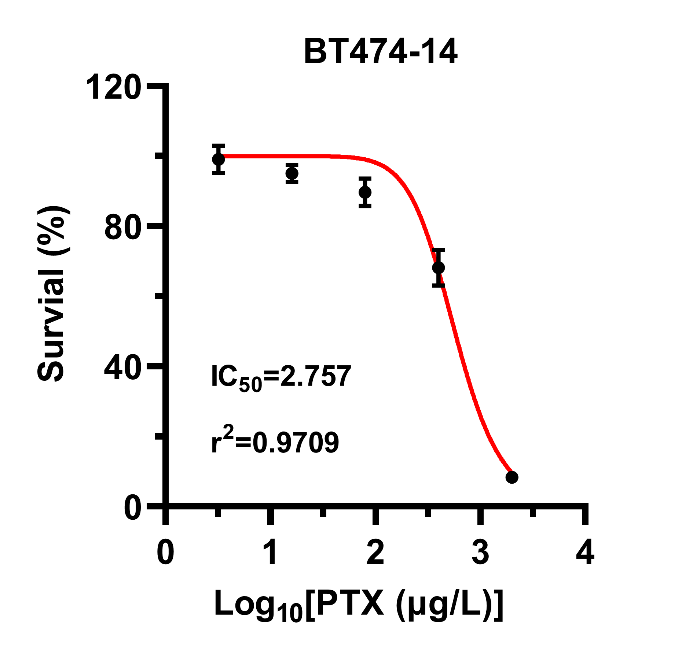

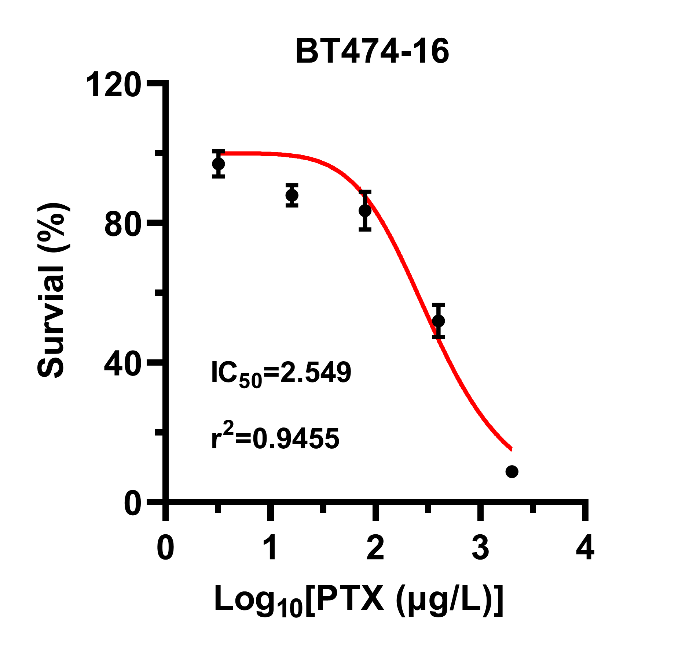


**Additional file 1: Figure S1. SOX4 knockdown increases IC50 of paclitaxel in BT474**.

Supplement: Supplementary file 1 — Additional file 1: Figure S1. SOX4 knockdown increases IC50 of paclitaxel in BT474. [file 12935_2020_1568_MOESM1_ESM.docx]
